# Rapid-Onset Acute Respiratory Distress Syndrome (ARDS) in a Patient Undergoing Metastatic Liver Resection: A Case Report and Review of the Literature

**DOI:** 10.1155/2010/586425

**Published:** 2010-08-15

**Authors:** Thorsten Brenner, Johann Motsch, Jens Werner, Lars Grenacher, Eike Martin, Stefan Hofer

**Affiliations:** ^1^Department of Anesthesiology, Heidelberg University, Im Neuenheimer Feld 110, 69120 Heidelberg, Germany; ^2^Department of Surgery, Heidelberg University, Im Neuenheimer Feld 110, 69120 Heidelberg, Germany; ^3^Department of Diagnostic Radiology, Heidelberg University, Im Neuenheimer Feld 110, 69120 Heidelberg, Germany

## Abstract

Metastatic liver resection following cytoreductive chemotherapy is an accepted treatment for oligometastatic tumor diseases. Although pulmonary complications are frequently reported in patients undergoing liver surgery including liver transplantation, life-threatening acute respiratory failures in the absence of aspiration, embolism, transfusion-related acute lung injury (TRALI), pulmonary infection, or an obvious source of systemic sepsis are rare. We performed an extensive clinical review of a patient undergoing metastatic liver resection who had a clinical course compatible to an acute respiratory distress syndrome (ARDS) without an obvious cause except for the surgical procedure and multiple preoperative chemotherapies. We hypothesize that either the surgical procedure mediated by cytokines and tumor necrosis factor or possible toxic effects of oxygen applied during general anesthesia were associated with life-threatening respiratory failure in the patient. Discrete and subclinical inflammated alveoli (probably due to multiple preoperative chemotherapies with substances at potential risk for interstitial pneumonitis as well as chest radiation) might therefore be considered as risk factors.

## 1. Case Report

**Figure 1:**
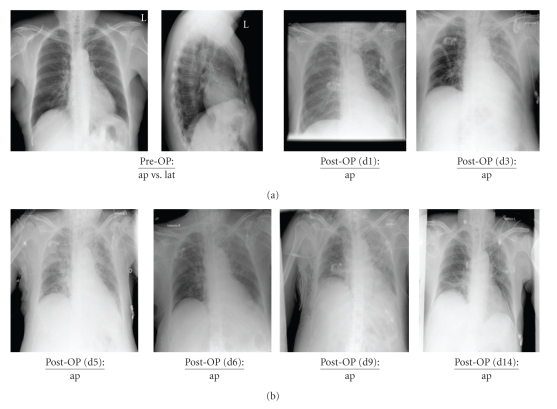
Chest X-ray diagnostic in the patient from preoperative (Pre-OP) until closely to the patient's discharge from hospital at day 14. Pre-OP: preoperative; d: day; ap: anterior-posterior; lat: lateral.

A 65-year-old, nonsmoking female patient (68 kg, 153 cm) was diagnosed with breast cancer (pT1c pN1b [2/25] cM0, G3, oestrogen receptor positive) of the left breast in the year 1994. In September 2009, the patient was presented for metastatic liver resection (S4a). The detailed history is presented in Table 1 whereas the patient's concomitant diseases and risk factors are presented in Table 2. The preoperative elevation revealed an unobtrusive lung function (VC, vital capacity: 2.31 L; FEV1, Forced expiratory vital capacity within 1 second: 2.18 L). In addition, preoperative chest X-ray showed no conspicuities (Figure 1). Accordingly, the patient was prepared for left-sided hemihepatectomy using a combined anesthesiological procedure (general anesthesia in combination with a thoracic epidural catheter). The first arterial blood gas analysis revealed a sufficient pulmonary function without any indication for an upcoming pulmonary complication (FIO_2_ 0.6, PaO_2_ 308.5 mmHg, PaCO_2_ 42.9 mmHg, HCO_3_ 32.9 mmol/L, BE 8.8 mmol/L). Approximately 90 minutes after induction of general anesthesia during the surgical phase of ongoing liver preparation, a stepwise decrease of the peripheral oxygen saturation (minimum 94%) under constant ventilator settings (FIO_2_ 0.44, VR 12/min, VT 450 mL, PEEP 3 mbar) became evident. Pulmonary auscultation revealed two-sided ventilation of the lungs with discrete inspiratory rales on both sides. Furthermore an arterial blood gas analysis was performed, showing a significantly decreased arterial oxygen partial pressure (PaO_2_ 68.8 mmHg). Under a stepwise increase of the inspired oxygen fraction up to FIO_2_ 1.0, the following measures were performed, but only led to short-term improvements.

- Open lung ventilation (Recruitment Maneuver).
- Lungs were sucked off (small amounts of a clear secretion).
- Diuretics were applied due to an insufficient diuresis in the initial phase of the surgical procedure.
- Prophylactic antiallergic therapy (single shot of 500 mg prednisolone, antihistaminic blockade).

Beside pulmonary problems, the patient further revealed instability of the circulatory system requiring an increased vasopressor and fluid administration during the surgical phase of liver resection. A further arterial blood gas analysis confirmed persisting hypoxemia (FIO_2_ 1.0; PaO_2_ 86.3 mmHg) in combination with slightly increasing PaCO_2_-values (PaCO_2_ 45.4 mmHg) under constant endexpiratory CO_2_-values (PetCO_2_ 33 mmHg). A transoesophageal echocardiography was performed immediately after an accelerated end of the surgical procedure. Beside the preexisting combined aortic valve defect (stenosis > insufficiency), no other newly developed valve defects such as pulmonary insufficiency or tricuspid valve insufficiency became obvious. Above all, both ventricles showed a normal configuration, without any signs of ventricular dysfunction.

**Figure 2:**
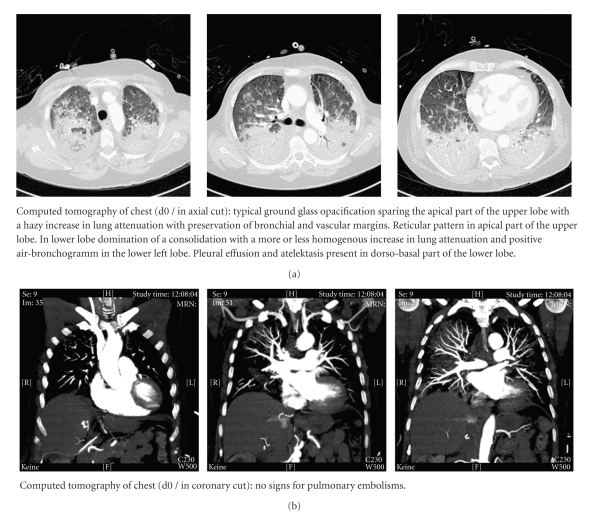
Coronary and axial cuts of the computed tomography of the chest immediately after the end of surgery before patient's admission to the intensive care unit. d: day.

Due to a progressing deterioration of the patient's cardiocirculatory and pulmonary conditions, in order to achieve further information a computed tomography of the chest was performed. The tomography of the chest showed typical signs for an acute respiratory distress syndrome (ARDS) whereas no signs for a pulmonary embolism were detected (Figure 2). After admission to the ICU, the respiratory situation remained critical, so the inhalation of nitric oxide was started. In addition, the patient received a high-dose corticosteroid regime (hydrocortisone 600 mg/24 h) for 5 days. No signs for an infection became obvious, as assessed by low-peak plasma levels of c-reactive protein (88.5 mg/L) and procalcitonin (0.06 ng/mL) one day after ARDS-onset. Furthermore, during initial ICU-stay microbiological investigations (tracheal secretions, hemoculture) did not reveal any sign for an infectious procedure.

**Figure 3:**
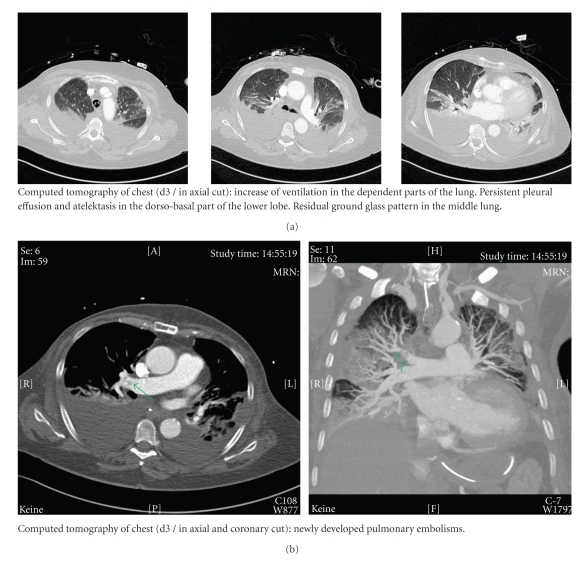
Coronary and axial cuts of the computed tomography of the chest at day 3 after onset of ARDS. d: day; ARDS: acute respiratory distress syndrome.

The following days an attempt was made to avoid an excessive fluid management. Prone positioning or surfactant replacement were discussed as therapeutical options but not realized due to a sufficient advancement of the respiratory conditions within the first hours after corticosteroid administration. Therefore, therapy with inhaled nitric oxide could already be finished eight hours after ARDS-onset. Despite a perioperative thrombosis prophylaxis on the ICU (UFH 10.000 IU/24 h), a newly developed pulmonary embolism (without a hemodynamic relevance) could be diagnosed by a computer tomography scan three days after ARDS-onset. As a result from this point on the patient received a full anticoagulation with UFH (25.000 IU/24 h). Duplex sonography was negative for deep vein thrombosis. Apart from this pulmonary embolism, as well as an increasing pleural effusion with the need for a chest tube, the weaning procedure was successful (Figure 3). Four days after ARDS-onset, the first attempt for an extubation was performed. Furthermore, corticosteroid therapy was reduced to the patient's preoperative corticosteroid application scheme due to rheumatoid arthritis (prednisone 5 mg/die).

Unfortunately, 24 hours after the initial extubation (5 days after ARDS-onset) the patient provided a respiratory deterioration with the need for a reintubation accompanied by a sudden increase of c-reactive protein and leucocytes. In response, an antibiotic therapy was initiated and the central venous catheter was changed. Afterwards the patient revealed a rapid amelioration of the respiratory conditions; at the same time plasma levels of c-reactive protein and leucocytes declined. The next attempt for an extubation was delayed due to an inadequate awakening. Finally, extubation was performed successfully 10 days after ARDS-onset. In the next days, the patient showed a favorable outcome. Counting from ARDS-onset, she was eligible for the transfer to intermediate care after 13 days and for her discharge from hospital after 18 days.

## 2. Discussion

Perioperative deterioration of pulmonary function represents a potentially life-threatening complication with the need for a fast and trigger-based therapy. Many triggers have to be taken into account in the case of acute perioperative lung failure.

### 2.1. Dislocation of the Endotracheal Tube/Pneumothorax

Dislocation of the endotracheal tube, as well as diaphragmatic lesions in the course of surgery leading to a pneumothorax can result in acute perioperative deterioration of the respiratory system. Especially surgical procedures in the upper abdominal area (e.g., surgery of the liver, kidneys or the spleen) are known to be at high risk for diaphragmatic injuries [1]. In both cases, a one-sided breath sound can be anticipated. Due to both-sided breath sounds in our patient as assessed by a repeated and intensive auscultation of the chest, as well as the absence of a diaphragmatic lesion, tube dislocation and pneumothorax could be excluded.

### 2.2. Aspiration

1.4–5.0 per 10.000 patients undergoing general anesthesia sustain a perioperative aspiration with a morbidity of 0.6–1.0 per 10.000 and a letality of 1.0–2.2 per 100.000 anesthesiological procedures. In total 10%–30% of anesthesia associated deaths can be attributed to complications of aspiration syndrome [2–4]. Especially patients undergoing emergency surgical procedures, as well as patients with gastric contents or other predisposing risk factors (e.g., obesity, reflux disease, etc.) are at high risk for perioperative aspiration. Due to an unobtrusive risk profile, a problem-free orotracheal intubation procedure (no prolonged phases of mask ventilation with the risk of gastric air insufflation, continuous cuff pressure levels between 25–30 cmH_2_O at all times) as well as successful placement of a stomach tube, aspiration as the main cause of acute respirators failure remains less likely.

### 2.3. Atelectasis

Due to a low-PEEP-ventilation regime, patients undergoing liver resections are at high risk for atelectatic alterations of the lungs [1]. Therefore a Recruitment Maneuver was performed after the onset of pulmonary deterioration. Afterwards a best-PEEP-ventilation regime according to the ARDS SepNet-Protocol was continued. Unfortunately, none of these efforts was able to optimize the patient's oxygenation. Therefore, the diagnosis of atelectasis-associated respiratory failure also is less likely.

### 2.4. Pulmonary Embolism

According to decreased central venous pressure as an effect of a restrictive fluid management and a low-PEEP-ventilation regime, surgical procedures involving the liver may be at high risk for embolisms (gas, tumor, etc.) [5, 6]. Furthermore, the clinical symptoms “severe hypoxemia, increasing PaCO_2_ values, and progressive hemodynamic instability” support the hypothesis of pulmonary embolism. Unfortunately, this hypothesis had to be rejected, since a transoesophageal echocardiography revealed no signs for a right ventricular dilatation/dysfunction as well as no newly developed valve defects such as pulmonary insufficiency or tricuspid valve insufficiency.

### 2.5. Cardiac Ischemia, Left Atrial Hypertension, Pulmonary Edema

Perioperative cardiac ischemia represents one of the most important risk factors for increased morbidity and mortality in the perioperative setting [7, 8]. Depending on individual risk factors, the intended surgical procedure as well as the underlying diagnostic criteria, the incidence of perioperative cardiac ischemia ranges from 4.5% to 78.0% and can mainly be observed in the early postoperative phase [8–14]. Therefore, perioperative left-ventricular ischemia leading to a left-sided cardiac dysfunction may be considered as a possible trigger for acute respiratory failure in patients undergoing major abdominal surgery. Due to a negative patient history for coronary artery disease and lack of echocardiographic signs for cardiac ischemia (regional disturbances of cardiac wall movements, left atrial hypertension), the diagnosis of pulmonary edema due to a left ventricular dysfunction became unlikely.

### 2.6. Transfusion Associated Lung Injury (TRALI)

With an incidence ranging from 1 : 2.000 to 1 : 5.000, transfusions of plasma containing blood products are known to be associated with the development of an acute lung injury due to donor-specific antibodies against granulocytes of the host [15]. It is defined as acute noncardiac hypoxemia/breathlessness in association with newly developed bilateral lung infiltrates within 6 hours after the transfusion. Because our patient did not receive any plasma containing blood products, the diagnosis of TRALI had to be rejected.

### 2.7. Transfusion Associated Circulatory Overload (TACO)

Acute hypoxemia, breathlessness, and tachypnea also appear as characteristic clinical signs for a TACO due to the development of a hydrostatic pulmonary edema within 6 hours following excessive perioperative volume overload [16, 17]. To distinguish between TRALI and TACO can cause some difficulties. In case of a TACO increasing levels of central venous pressure and pulmonary arterial pressure, a tachycardia as well as a hypertonic derailment can be observed [16, 17]. In the literature, the incidence of TACO is reported to be between 1 : 356 and 1 : 3.000. Especially patients with preexisting cardiac diseases (e.g., cardiac insufficiency or coronary artery heart disease) seem to be at risk. Due to negative history for active cardiac conditions, a restrictive perioperative volume replacement regime without any transfusions as well as a normal transoesophageal echocardiography, the diagnosis of a TACO also had to be rejected.

Unfortunately, despite intensive diagnostics, the reason for respiratory failure in this patient remains unknown. Therefore, a computed tomography of the chest was performed. Although the diagnosis of an acute respiratory distress syndrome (ARDS) was pointed out by the radiologist, the inducing trigger further remained unknown.

Some of the disorders associated with ARDS such as presented in Table 3 (e.g., aspiration, transfusion associated lung injury, embolism, etc.) were already excluded at bedside, so that three further triggers were now critically discussed.

### 2.8. Sepsis, Pneumonia

Infection-related disorders such as severe sepsis and/or septic shock are frequently associated with the occurrence of an ARDS. The SepNet trial revealed a prevalence of severe sepsis/septic shock of 11% and a resulting mortality of 48% on intensive care units. Therefore, severe sepsis/septic shock are the most common causes of death in noncardiologic intensive care medicine. In most cases, an infection of the respiratory tract (63%) or an intraabdominal infection (25%) is found to be the focus [18]. According to the ACCP/SCCM-Consensus Conference, sepsis is defined by the presence of both infection (documented or suspected) and a systemic inflammatory response [19]. Due to low peak plasma levels of c-reactive protein and procalcitonin, a normal leucocyte count as well as negative microbiological investigations (tracheal secretion, hemoculture) in the pre- and postoperative phase, an infectious ARDS-focus (e.g., pneumonia) could not be identified.

### 2.9. Respiratory Burst

Several chemotherapeutics such as docetaxel, trastuzumab, lapatinib, gemcitabine, cisplatin, busulfan/melphalan, bleomycin, and so forth, seem to be associated with the development of a severe pneumonitis leading to ARDS even though it represents a very rare side effect [20–30]. 

The patient presented in this case report received docetaxel, trastuzumab, as well as lapatinib; accordingly the patient was treated with potential pneumonitis inducing agents. In addition, a chest radiation was performed twice. In patients at high risk for preexisting inflammatory changes of the lung, for example, pneumonitis or alveolitis, especially the induction of general anesthesia using elevated inspired oxygen fraction concentrations is known to contribute to the onset of an acute respiratory distress syndrome (ARDS). After induction, the preexisting endothelial dysfunction causes cells and inflammatory exudates to enter the alveoli with a concomitant increase of the thickness of the alveolo-capillary space, leading to hypoxia and secondary induction of fibrosis of the airspace. In contrast to patients with hydrostatic pulmonary edema, in ARDS patients' fluid removal from the alveolar space is impaired [31, 32]. Furthermore, in patients with ARDS surfactant synthesis and function are also reduced leading to decreased lung compliance [33–36]. 

In our patient, we suspected as a precursor for ARDS-onset an acute exacerbation of a preexisting subclinical pneumonitis. In contrast to the existing literature, our patient developed symptoms of ARDS as early as two hours after the induction of general anesthesia, although the pulmonary status before and immediately after induction of general anesthesia revealed no sign for an impairment as assessed by a preoperative X-ray, preoperative spirometrical investigation, and an arterial blood gas analysis after the induction of general anesthesia. The rapid improvement of lung oxygenation following corticosteroid administration and the lack of bacterial, viral, or fungal infections of the lungs may support our hypothesis of a hypersensitivity-pneumonitis-based-ARDS with a good response to anti-inflammatory therapeutic strategies.

### 2.10. Tumor-Associated Cytokine Storm

In the literature, cases of ARDS following cytoreductive surgery have been described [37, 38]. Eltabbakh and colleagues report of a 58-year-old woman who developed ARDS following extensive cytoreductive surgery for a widely disseminated intraperitoneal leiomyosarcoma. It was concluded that the onset of ARDS might be due to the massive cytoreductive surgery probably mediated by cytokines and tumor necrosis factor. Furthermore, multiple transfusions were discussed as potential ARDS-inductors [38]. Alonso and colleagues presented two patients who had a clinical course compatible with ARDS without obvious cause except for the cytoreductive surgery, perioperative intraperitoneal as well as intrapleural chemotherapy. These two patients developed gradually increasing respiratory distress in the postoperative period. No bacterial or fungal infections of lungs or intra-abdominal sites or sepsis were discovered. It was concluded that aggressive cytoreductive surgery along with perioperative chemotherapy was associated with life-threatening respiratory failure in these two patients.

However, a left-sided hemihepatectomy as performed in our patient cannot be described as an extensive cytoreductive surgery. The authors hypothesize that beside the amount of cytoreduction, the perfusion of the manipulated tissue might play an important role. The liver is known as one of the organs with the best perfusion. About 25% of the heart time volume circulates continuously through the liver [39]. Therefore, liberation of cytokines and tumor necrosis factor caused by surgical manipulations of tumorous liver tissue during the preparation phase may lead to a rapid systemic dissemination. After liberation of the cytokines into the bloodstream, the lung represents one of the first organs getting in contact with these cytokines, resulting in disseminated inflammation. This effect might be perpetuated by the preoperative application of multiple chemotherapeutics, probably associated with an inflammatory preconditioning of the lungs.

Conditions that initiated the sequences of events that resulted in ARDS in our patient were not clearly associated with the clinical conditions causing the syndrome as described in the medical literature. Nevertheless, after excluding most common other disorders associated with ARDS (Table 3), the two disorders finally discussed here remained the very likely causes for ARDS-induction in the patient. At the same time, it still remains unclear whether one of these pathways alone or a combination of both contributed to the rapid onset of ARDS in our patient.

**Figure 4:**
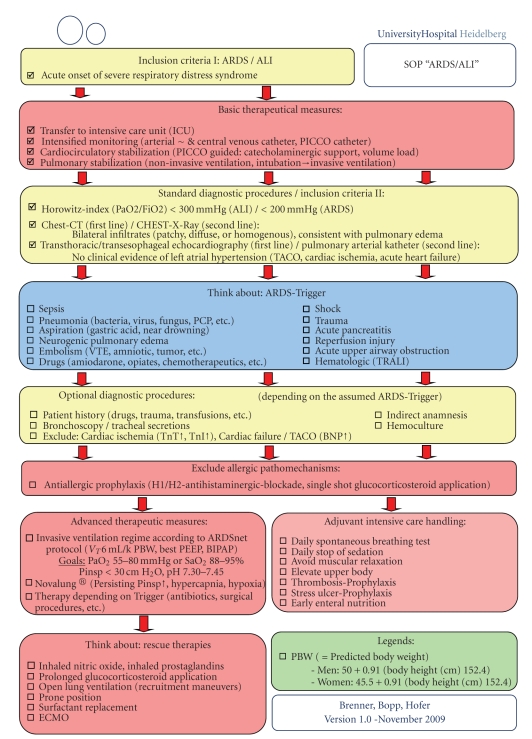
Standard operating procedure (SOP) of the Department of Anesthesiology, University of Heidelberg, Germany, for the diagnosis and management of patients with suspected ARDS.

Additionally, as a result of this case and the different, in some parts, controversially discussed therapeutical options available for ARDS, we introduced a new algorithm (standard operating procedure (SOP) of the Department of Anesthesiology (University of Heidelberg, Germany)) for the diagnosis and management of patients with suspected ARDS (Figure 4).

## 3. Conclusions

Patients with preoperative cytoreductive chemotherapy using potentially pneumonitis-inducing agents prior to the surgical procedure should be intensively monitored concerning their pulmonary function under general anesthesia. First, strongly elevated inspired oxygen fraction concentrations may be a potential risk for an “inflammatory burst” of immune cells in the lung leading to an ARDS and should, therefore, be avoided whenever possible. Secondly, one needs to reduce to a minimum the surgeon's contribution to the ARDS-onset through intensive tumor manipulations prior to resection resulting in massive cytokine liberation.

##  Competing Interests

The authors declare that they have no competing interests.

## Figures and Tables

**Table 1:** Anamnesis.

December 1994	Diagnosis of breast cancer followed by a resection of the left breast
January 1995–June 1995	Adjuvant chemotherapy (cyclophosphamide, metothrexate, 5-fluoruracil)
January 1995–June 2005	Tamoxifen
June 2005	Diagnosis and extirpation of a lymph node metastasis in the left supraclavicular area
July 2005–September 2005	Radiation of the left supraclavicular area
December 2005	Diagnosis of a metastasis in the ventral part of the mediastinum with an osteolytic destruction of the breastbone
January 2006–February 2006	Radiation of the mediastinum
March 2006	Diagnosis of a liver metastasis (S4a)
March 2006–July 2006	Chemotherapy (trastuzumab, docetaxel) was followed by a complete remission of the metastasis
July 2006–October 2008	Monotherapy with trastuzumab
October 2008	Reappearance of the liver metastasis with a dimension increase
October 2008–March 2009	Chemotherapeutics were escalated towards lapatinib and capecitabine
March 2009–August 2009	Dimension decrease of the liver metastasis. Monotherapy with lapatinib due to capecitabine associated hemorrhagic diarrhea.
September 2009	Left-sided hemihepatectomy followed by an ARDS

**Table 2:** The patient's concomitant diseases.

Essential arterial hypertension
Combined aortic valve defect (stenosis > insufficiency)
Essential hyperlipoproteinemia
Rheumatoid Arthritis under corticosteroid treatment (5 mg prednisone/die)
Diabetes mellitus II (Insulin-dependent)
Nodular goiter (euthyroid)

**Table 3:** Disorders associated with the acute respiratory distress syndrome (ARDS).

Sepsis (most common)
Aspiration
Pneumonia (bacterial, viral, fungal, etc.)
Embolism (VTE, amniotic, tumor, etc.)
Hematologic (transfusion-related acute lung injury, TRALI)
Shock (any etiology)
Trauma
Acute pancreatitis
Drugs (amiodarone, tocolytics, salicylates, opiates, etc.)
Reperfusion injury (post-lung transplant, lung reexpansion)
Acute upper airway obstruction
Neurogenic pulmonary edema

## Abbreviation List

ALI: Acute lung injury
ARDS: Acute respiratory distress syndrome
BNP: Brain natriuretic peptide
CT: Computed tomography
ECMO: Extracorporeal membrane oxygenation
FEV1: Forced expiratory vital capacity within 1 second
ICU: Intensive care unit
IU: International units
NSCLC: Non-small-cell lung cancer
PBW: Predicted body weight
PEEP: Positive endexpiratory pressure
PICCO: Pulse contour cardiac output
SOP: Standard operating procedure
TACO: Transfusion associated cardiac overload
TnI: Troponine I
TnT: Troponine T
TRALI: Transfusion-related acute lung injury
UFH: Unfractionated heparin
VC: Vital capacity
VR: Ventilator rate
VT: Tidal volume.
